# Final results of a phase 1b study of the safety and efficacy of the PI3Kδ inhibitor acalisib (GS-9820) in relapsed/refractory lymphoid malignancies

**DOI:** 10.1038/s41408-018-0055-x

**Published:** 2018-02-12

**Authors:** Arnon P. Kater, Sanne H. Tonino, Marjolein Spiering, Martine E. D. Chamuleau, Roberto Liu, Adeboye Henry Adewoye, Jie Gao, Lyndah Dreiling, Yan Xin, Jeanette K. Doorduijn, Marie José Kersten, on behalf of the HOVON Lunenburg Lymphoma Phase I/II Consortium

**Affiliations:** 1 Department of Hematology, Academic Medical Center, Amsterdam, The Netherlands; 2 Lymphoma and Myeloma Center (LYMMCARE), Academic Medical Center, Amsterdam, The Netherlands; 3 Department of Hematology, VU University Medical Center, Amsterdam, The Netherlands; 4 Clinical Trial Office Department of Hematology, Academic Medical Center, Amsterdam, The Netherlands; 5 Gilead Sciences Inc., Foster City, CA USA; 6 Department of Hematology, Erasmus MC Cancer Institute, Rotterdam, The Netherlands

Chronic lymphocytic leukemia (CLL) and B-cell lymphomas represent a heterogeneous group of common hematologic malignancies arising as a consequence of dysregulated B-cell differentiation and expansion of an abnormal B-cell clone^1^. Due to an unmet clinical need in elderly patients with relapsed/refractory B-cell malignancies for whom currently there are no curative options^2,3^, we evaluated the clinical activity and safety of acalisib (GS-9820, 6-fluoro-3-phenyl-2-[(1S)-1-(9H-purin-6-ylamino) ethyl]-4(3H)-quinazolinone), a second-generation inhibitor of phosphoinositide 3-kinase delta (PI3Kδ).

In a human basophil activation assay, acalisib was most selective for PI3Kδ (IC_50_ 12.7 nM), compared with other PI3K class I isoforms. Acalisib suppressed IgE receptor I PI3Kδ-mediated CD63 expression with an EC_50_ of 14 nM. Its high selectivity was confirmed by the fact that no binding to other kinases was observed at 10 µM acalisib concentration^4^.

We initiated a phase 1b, open-label, dose escalation and expansion study of acalisib monotherapy in adults with recurrent lymphoid malignancies, who had measurable lymphadenopathy and required therapy for their cancers (NCT01705847). Eligible patients were enrolled into escalating dose cohorts utilizing a 3 + 3 design at the following oral acalisib doses: 50, 100, 200, and 400 mg twice daily. The primary endpoint of the study was to evaluate the maximum tolerated dose (MTD) within the tested dose range. The secondary study objectives were to characterize the dose-limiting toxicities, efficacy (per standard response criteria^5,6^), and overall safety profile of acalisib (details in Supplemental Materials).

From 21 November 2012 to 30 April 2014, 39 patients were enrolled in four centers in the Netherlands. The final data cutoff presented herein is 17 August 2016, with a median (min, max) follow-up of 6 (0.03, 37.0) months. In total, 38 patients received ≥1 dose of acalisib—four, three and three patients in the acalisib 50, 100, and 200 mg twice-daily dose-finding cohorts, respectively, and 29 patients in the 400 mg twice-daily expansion cohort. Thirty-four (89.5%) patients discontinued prior to study closure, primarily due to progressive disease (12 (32%)) (Supplemental Table 1).

The majority of patients were male 26/38 (68%), with a median age (range) of 69 (48–81) years and a median (Q1, Q3) number of 3 (2, 4) prior therapies. Overall, 22 (57.9%) patients had CLL, 15 (39.5%) had non-Hodgkin’s lymphoma (NHL) and 1 (2.6%) had (Hodgkin’s lymphoma (HL); >50% had refractory disease and 34.2% had relapsed after the last therapy (Supplemental Table 2).

After a median treatment duration of 5.8 months, the overall response rate (ORR) (95% confidence interval (CI)) in all dose cohorts per independent review committee assessment was 42.1% (26.3, 59.2) with 16 partial responses (PRs) (Supplemental Table 3). Responses were observed across all dose cohorts (Supplemental Fig. 1). The ORR (95% CI) for the 29 patients who received 400 mg acalisib twice daily as their initial dose level was 41.4% (23.5, 61.1), with 12 PRs (Supplemental Table 3). When efficacy was analyzed by disease type, a higher ORR (95% CI) was noted in patients with CLL (53.3% (26.6, 78.7)), compared with NHL/HL patients (28.6% (8.4, 58.1)) (Supplemental Table 3).

The lymph node response (95% CI) was 12/14 (85.7% (57.2, 98.2)) in patients with CLL and 4/11 (36.4% (10.9, 69.2)) in patients with NHL/HL. Among patients with CLL, the best percent change from baseline in the sum of the products of the greatest perpendicular diameters exceeding 50% was observed in all dose cohorts, whereas patients with NHL responded only to the highest dose of acalisib (Fig. 1).

**Fig. 1:**
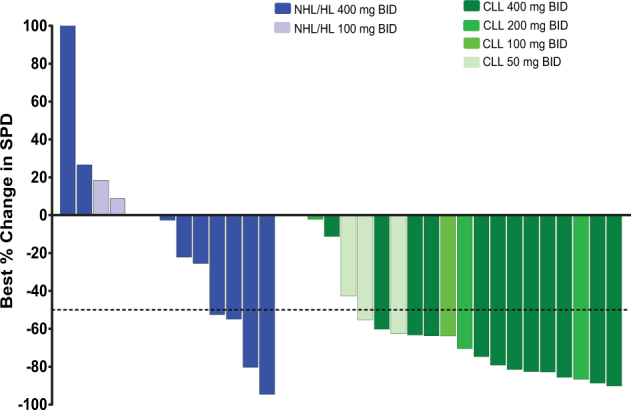
Best percent change from baseline in the SPD per disease type and IRC assessments^a^. This analysis includes patients with both baseline and post-baseline SPD measurements. Baseline is defined as the last measurement before the first dose of GS-9820. ^a^Patients were analyzed based on the initial dosing level they received. CLL chronic lymphocytic leukemia, NHL/HL non-Hodgkin’s and Hodgkin’s lymphoma, SPD sum of the products of the greatest perpendicular diameters

After a median (Q1, Q3) follow-up of 4.5 (2, 16) months, the median (95% CI) Kaplan–Meier estimate of PFS for the 29 patients initially treated with 400 mg acalisib twice daily was 8.2 (3.4, 16.6) months; 16.6 (3.4, NR) months for patients with CLL and 4.0 (1.6, 16.4) months for patients with NHL/HL (Supplemental Fig. 2).

The median (Q1, Q3 (range)) duration of exposure to acalisib was 5.8 (2, 17 (0–37)) months for all 38 treated patients, and 4.5 (2, 16 (0–30)) months for the 29 patients who received 400 mg twice daily as their initial treatment. Doses ranging from 50 to 400 mg twice daily were not associated with dose-limiting toxicities (DLTs) and the MTD dose has not been determined.

Adverse events (AEs, not exposure-adjusted) were reported in all treated patients (Supplemental Table 4–6) and included all AEs, laboratory abnormalities, and serious AEs. Treatment-emergent AEs (TEAEs) considered related to acalisib were reported by 30 (78.9%) patients, of whom 21 (55.3%) had grade ≥3 events. The most frequent TEAEs were diarrhea, rash, elevated liver transaminases, and infections (Table 1). Grade ≥3 infections (pneumonia, viral pneumonia, bronchiolitis, and *Pneumocystis jirovecii* pneumonia) were reported individually in four patients. Although at the time of the first interim analysis performed in July 2013 none of the patients had grade ≥3 elevations of hepatic transaminase levels, extended duration of treatment led to 10.5% and 7.9% of patients with grade ≥3 laboratory alanine aminotransferase (ALT) and aspartate aminotransferase (AST) elevations, respectively. After dose interruption, 7.9% of these events resolved to grade 1.

**Table 1:** Adverse events related to acalisib in ≥5% of all patients, and all acalisib-related infections

Adverse events, *n* (%)	50 mg BID (*N* = 3)		100 mg BID (*N* = 3)		200 mg BID (*N* = 3)		400 mg BID (*N* = 29)		Total (*N* = 38)	
	All	Grade ≥3	All	Grade ≥3	All	Grade ≥3	All	Grade ≥3	All	Grade ≥3
AEs related to acalisib	3 (100)	1 (33.3)	2 (66.7)	1 (33.3)	2 (66.7)	1 (33.3)	23 (79.3)	18 (62.1)	30 (78.9)	21 (55.3)
Diarrhea	2 (66.7)	1 (33.3)	0	0	1 (33.3)	0	6 (20.7)	3 (10.3)	9 (23.7)	4 (10.5)
Rash	0	0	0	0	0	0	8 (27.6)	4 (13.8)	8 (21.1)	4 (10.5)
Weight decreased	1 (33.3)	0	0	0	0	0	5 (17.2)	1 (3.4)	6 (15.8)	1 (2.6)
Alanine aminotransferase increased	0	0	1 (33.3)	0	0	0	4 (13.8)	1 (3.4)	5 (13.2)	1 (2.6)
Aspartate aminotransferase increased	0	0	1 (33.3)	0	0	0	4 (13.8)	1 (3.4)	5 (13.2)	1 (2.6)
Dysgeusia	0	0	0	0	0	0	5 (17.2)	0	5 (13.2)	0
Cough	0	0	0	0	0	0	4 (13.8)	0	4 (10.5)	0
Pyrexia	0	0	0	0	0	0	3 (10.3)	0	3 (7.9)	0
Nausea	0	0	0	0	0	0	3 (10.3)	0	3 (7.9)	0
Neutrophil count decreased	0	0	0	0	0	0	3 (10.3)	3 (10.3)	3 (7.9)	3 (7.9)
Drug eruption	0	0	0	0	0	0	2 (6.9)	0	2 (5.3)	0
Blood alkaline phosphatase increased	0	0	0	0	0	0	2 (6.9)	0	2 (5.3)	0
Anemia	0	0	0	0	0	0	2 (6.9)	1 (3.4)	2 (5.3)	1 (2.6)
Hypokalemia	0	0	0	0	0	0	2 (6.9)	1 (3.4)	2 (5.3)	1 (2.6)
All acalisib-related infections and infestations, by preferred term										
Pneumonia	1 (33.3)	0	0	0	0	0	1 (3.4)	1 (3.4)	2 (5.3)	1 (2.6)
Lung infection	0	0	0	0	0	0	1 (3.4)	0	1 (2.6)	0
Bronchiolitis	0	0	0	0	0	0	1 (3.4)	1 (3.4)	1 (2.6)	1 (2.6)
Pneumonia viral	0	0	0	0	0	0	1 (3.4)	1 (3.4)	1 (2.6)	1 (2.6)
Mycobacterial infection	0	0	0	0	0	0	1 (3.4)	0	1 (2.6)	0
Varicella zoster virus infection	0	0	0	0	0	0	1 (3.4)	0	1 (2.6)	0
Parainfluenza virus infection	0	0	0	0	0	0	1 (3.4)	0	1 (2.6)	0
Pneumocystis jirovecii pneumonia	0	0	0	0	0	0	1 (3.4)	1 (3.4)	1 (2.6)	1 (2.6)
Upper respiratory tract infection	0	0	0	0	0	0	1 (3.4)	0	1 (2.6)	0
Urinary tract infection	0	0	0	0	1 (33.3)	1 (33.3)	0	0	1 (2.6)	1 (2.6)

Patients who experienced multiple events within the same preferred term are counted once

*BID* twice daily

Acalisib-related AEs that led to permanent discontinuation from the study drug occurred in the acalisib 400 mg twice-daily cohort only, and included: grade 5 leukoencephalopathy, grade 2 exanthema, grade 2 allergic reaction (rash), grade 3 hemolysis, grade 3 hypersensitivity allergic reaction, and grade 3 ALT and AST elevations.

In the overall study population, nine (23.7%) patients had an AE that led to death—these included two patients with pneumonia, two with respiratory failure, and one patient each with dyspnea, general physical health deterioration, leukoencephalopathy, pulmonary sepsis, and tumor embolism. Among these nine patients, one patient was in the 50 mg acalisib twice-daily cohort, and the remaining eight patients were treated with 400 mg acalisib twice daily.

In conclusion, acalisib demonstrated clinical activity in the study population. The prominent TEAEs were due to a spectrum of mostly infectious and immune-mediated toxicities described for other PI3Kδ inhibitors (idelalisib, duvelisib)^7,8^, which may point to a class effect rather than a specific drug effect^9–11^.

## Electronic supplementary material

Supplemental Materials
