# Supplementary material for: Final results of a phase 1b study of the safety and efficacy of the PI3Kδ inhibitor acalisib (GS-9820) in relapsed/refractory lymphoid malignancies
Source: Blood Cancer J. 2018 Feb 12;8(2):16. doi: 10.1038/s41408-018-0055-x (PMC5809482; doi:10.1038/s41408-018-0055-x)
Supplement: Supplementary file 1 — Supplemental Materials [file 41408_2018_55_MOESM1_ESM.pdf]

Supplemental Materials

Final results of a phase 1b study of the safety and efficacy of the PI3Kδ inhibitor acalisib (GS-9820) in relapsed/refractory lymphoid malignancies

Arnon P. Kater[1,2], Sanne H. Tonino[1,2], Marjolein Spiering[1], Martine E. D. Chamuleau[3], Roberto Liu[4], Adeboye Henry Adewoye[5], Jie Gao[5], Lyndah Dreiling[5], Yan Xin[5], Jeanette K. Doorduijn[6], and Marie José Kersten[1,2] on behalf of the HOVON Lunenburg Lymphoma Phase I/II Consortium

[1]Department of Hematology, and [2]Lymphoma and Myeloma Center (LYMMCARE), Academic Medical Center, Amsterdam, the Netherlands

[3]Department of Hematology, VU University Medical Center, Amsterdam, the Netherlands

[4]Clinical Trial Office Department of Hematology, Academic Medical Center, Amsterdam, the Netherlands

[5]Gilead Sciences Inc., Foster City, CA, USA

[6]Department of Hematology, Erasmus MC Cancer Institute, Rotterdam, the Netherlands

Methods

Supplemental Table 1. Patient distribution

Supplemental Table 2. Patient demographic and baseline characteristics

Supplemental Table 3. ORR per IRC assessment.

Supplemental Table 4. All grade and grade ≥3 adverse events in ≥10% of patients

Supplemental Table 5. Laboratory abnormalities reported in ≥15% of patients

Supplemental Table 6. Serious adverse events in ≥5% of all patients and all serious infections

Supplemental Figure 1. Forest plot of overall response rate per dose cohort and IRC assessment

Supplemental Figure 2. Kaplan-Meier curve of progression-free survival per disease type and IRC assessment in 29 patients treated with acalisib 400 mg twice daily

Methods

Study design and treatments

Eligible patients were enrolled into escalating dose cohorts utilizing a 3+3 design at the following oral acalisib doses: 50, 100, 200, and 400 mg twice daily. This study was approved by the Independent Ethics Committee and Institutional Review Board at each of the 4 centers in the Netherlands.

The primary endpoint was to establish the maximum tolerated dose (MTD), defined as the highest tested dose associated with a rate of dose-limiting toxicity (DLT) of <33% during the first 4 weeks of therapy. A DLT was defined as the occurrence of any acalisib-related grade 4 hematological toxicity without clinical sequelae (bleeding, infection) persisting for >7 days, or grade ≥3 toxicities of other types. Patients could be enrolled in the study to receive treatment at or below the MTD. If the MTD was not reached, additional patients could be enrolled at 1 or more dose levels to further evaluate the safety, efficacy, and pharmacokinetics (PK). Acalisib was taken continuously until disease progression, intolerable toxicity, or study discontinuation.

The secondary study objectives were to characterize the PK, efficacy, and overall safety of acalisib. Pharmacokinetic endpoints included plasma concentration over time, maximum plasma concentration, time to maximum plasma concentration, and area under the concentration-time curve of acalisib. The efficacy endpoints were overall response rate (ORR), time to response (TTR), duration of response (DOR), progression-free survival (PFS), and lymph node response (LNR). Responses were assessed by the independent review committee (IRC). The safety of acalisib doses was characterized by the type, frequency, severity, timing of onset, duration, and relationship to study therapy of any adverse events or laboratory test abnormalities.

Patients

Key inclusion criteria were: adult patients diagnosed with chronic lymphocytic leukemia (CLL), Hodgkin's lymphoma (HL), or non-Hodgkin's lymphoma (NHL)—including follicular lymphoma (FL), small lymphocytic lymphoma (SLL), lymphoplasmacytoid lymphoma/Waldenström's macroglobulinemia (LPL/WM) and marginal zone lymphoma (MZL)—and patients with diffuse large B-cell lymphoma (DLBCL) or mantle cell lymphoma (MCL). Histology was evaluated based on the classification established by the World Health Organization in 2008. To be eligible for enrollment, patients had to have ≥1 prior therapies, Karnofsky performance score of ≥60, and radiographically measurable lymphadenopathy or extranodal lymphoid malignancy (defined as the presence of ≥1 lesion that measures ≥2.0 cm in the longest dimension and ≥1.0 cm in the longest perpendicular dimension). Key exclusion criteria were patients with known Richter's transformation; active central nervous system or leptomeningeal lymphoma; myelodysplastic syndrome; ongoing, drug-induced pneumonitis or liver injury; and/or chronic active viral hepatitis. All enrolled patients provided written informed consent prior to study participation.

Assessments

Blood samples for PK evaluation were collected at predose and at 0.5, 1, 1.5, 2, 3, 4, and 6 hours after acalisib administration on day 1 (first dosing day) and on day 29 (steady state). Plasma concentrations of acalisib were analyzed using a validated liquid chromatography/mass spectrometry method with a lower limit of quantitation of 1 ng/mL. Pharmacokinetic parameters were generated using noncompartmental analysis in WinNonlin (version 6.3, Pharsight, Mountain View, California).

Antitumor activity was evaluated using standard response criteria for the tumor types in patients enrolled, with adjustment to account for the effects on lymphocytosis observed with B-cell receptor inhibitors. Patients were assessed for tumor response by computerized axial tomography and/or magnetic resonance imaging at weeks 8, 16, 24, 36, and 48; and every 12 weeks thereafter through week 96. The findings of the IRC were considered primary for analyses of all tumor control endpoints.

Clinic/laboratory visits occurred at 1- to 2-week intervals through week 24, every 6 weeks between weeks 24 and 48, and every 12 weeks thereafter. Descriptions of any AEs, DLTs, serious AEs, or AEs leading to discontinuation of study treatment were collected. Adverse events were coded according to the Medical Dictionary for Regulatory Activities (MedDRA) version 19.0.

Statistical analyses

The sample size for this phase 1 study was not based on a formal statistical hypothesis; in the dose-escalation phase, each dose cohort was 3 patients, and 3 additional patients could be included if 1 DLT was observed. The total planned number of patients enrolled in stage 1 was up to 40. The rationale for expanding the 400 mg twice daily cohort was to further evaluate the safety and efficacy of the highest dose tested. With the assumption of 50% ORR based on the observed data, enrolling 30 patients should provide 80% power to detect the true ORR larger than 30%, with a 1-sided test with significance level of 0.1. With the assumption of 44% ORR, the power would be 60% with 30 patients.

As this was a nonrandomized study, the full analysis set was the same as the safety analysis set and included all patients who received ≥1 dose of acalisib. The safety analysis set was used in the analyses of patient characteristics, efficacy, and safety.

Measures of antitumor activity included ORR, defined as the proportion of patients who achieved a complete response (CR) and partial response (PR); TTR, defined as the interval from start of study treatment to the first documentation of CR or PR; DOR, defined as the interval from the first documentation of CR or PR to the earlier of the first documentation of definitive disease progression or death from any cause; LNR, defined as the proportion of patients who achieved a ≥50% decrease from baseline in the sum of the products of the greatest perpendicular diameters (SPD) of index lymph nodes; and PFS, defined as the interval from the start of study treatment to the earlier of the first documentation of definitive disease progression or death from any cause.

Definitive disease progression for patients with CLL was based on standard criteria and included progressive disease (PD) occurring for any reason other than lymphocytosis alone (ie, increasing lymphadenopathy, organomegaly, or bone marrow involvement; decreasing platelet count, hemoglobin, or neutrophil count).

Patient baseline characteristics, demographics, and safety were summarized using descriptive statistics. For binary variables, 95% confidence intervals (CIs) were calculated using the binomial distribution, and using Greenwood's formula with (complementary) log-log transformation for Kaplan-Meier estimates.

Supplemental Table 1. Patient disposition by acalisib dose cohort[a, b]

	50 mg BID	100 mg BID	200 mg BID	400 mg BID	Total
**Number of patients, n (%)**					
Screened	4	3	3	34	44
Enrolled	4	3	3	29	38
Full analysis set	3 (100)	3 (100)	3 (100)	29 (100)	38 (100)
Safety analysis set	3 (100)	3 (100)	3 (100)	29 (100)	38 (100)
PK analysis set	3 (100)	3 (100)	3 (100)	27 (93)	36 (95)
Discontinued	3 (100)	3 (100)	3 (100)	29 (100)	38 (100)
Entered the 5-year follow-up	1 (33)	3 (100)	3 (100)	13 (45)	20 (53)
**Primary reason for study discontinuation, n (%)**					
Progressive disease	1 (33)	3 (100)	1 (33)	7 (24)	12 (32)
Adverse event	0	0	0	9 (31)	9 (24)
Death	2 (67)	0	0	6 (21)	8 (21)
Study discontinued by sponsor	0	0	2 (67)	4 (14)	6 (16)
Physician decision	0	0	0	3 (10)	3 (8)

[a]The full analysis set includes patients who received ≥1 dose of acalisib.

[b]Percentages are based on the number of subjects in the full analysis set.

BID, twice daily.

Supplemental Table 2. Patient demographic and baseline characteristics

	50 mg BID (N = 3)	100 mg BID (N = 3)	200 mg BID (N = 3)	400 mg BID (N = 29)	Total (N = 38)
**Sex, n (%)**					
Male	1 (33)	2 (67)	2 (67)	21 (72)	26 (68)
Female	2 (67)	1 (33)	1 (33)	8 (28)	12 (32)
**Race, n (%)**					
White	0	0	0	6 (21)	6 (16)
Not disclosed	3 (100)	3 (100)	3 (100)	23 (79)	32 (84)
**Age, years**					
Median	68.0	65.0	74.0	69.0	69.0
Q1, Q3	65.0, 71.0	54.0, 65.0	71.0, 81.0	59.0, 73.0	62.0, 73.0
**Karnofsky performance status, n (%)**					
100	2 (66.7)	2 (66.7)	2 (66.7)	19 (65.5)	25 (65.8)
>60–<100	0	0	1 (33.3)	8 (27.6)	9 (23.7)
60	1 (33.3)	1 (33.3)	0	2 (6.9)	4 (10.5)
**Disease history, n (%)**					
CLL	3 (100.0)	1 (33.3)	3 (100.0)	15 (51.7)	22 (57.9)
NHL	0	2 (66.7)	0	13 (44.8)	15 (39.5)
FL	0	1 (33.3)	0	0	1 (2.6)
LPL/WM	0	1 (33.3)	0	0	1 (2.6)
MZL	0	0	0	1 (3.4)	1 (2.6)
DLBCL	0	0	0	4 (13.8)	4 (10.5)
MCL	0	0	0	8 (27.6)	8 (21.1)
HL	0	0	0	1 (3.4)	1 (2.6)
**Relapse or refractory disease, n (%)**					
Relapsed	1 (33.3)	0	0	12 (41.4)	13 (34.2)
Refractory[a]	2 (66.7)	2 (66.7)	3 (100.0)	14 (48.3)	21 (55.3)
Unknown[b]	0	1 (33.3)	0	3 (10.3)	4 (10.5)

**Time since diagnosis, years**					
Median	8.0	3.4	3.2	4.5	4.8
Q1, Q3	6, 14	3, 10	1, 6	3, 9	3, 9
**Time since completion of last regimen, months**					
Median	3.3	2.6	3.6	3.6	3.5
Q1, Q3	3, 62	0, 17	1, 4	2, 9	2, 9
**Time since most recent relapse/refractory diagnosis, months**					
Median	3.5	3.5	4.3	4.9	4.7
Q1, Q3	3, 63	1, 17	1, 5	3, 13	3, 13
**Number of prior therapies**					
Median	4.0	4.0	3.0	3.0	3.0
Q1, Q3	1, 7	3, 6	2, 5	2, 4	2, 4
**Number of patients with prior therapies, n (%)**					
≥1–2	1 (33.3)	0	1 (33.3)	12 (41.4)	14 (36.8)
≥3–4	1 (33.3)	2 (66.7)	1 (33.3)	14 (48.3)	18 (47.4)
≥5–6	1 (33.3)	1 (33.3)	1 (33.3)	3 (10.3)	6 (15.8)
**Prior therapy type, n (%)**					
Rituximab	2 (66.7)	3 (100.0)	3 (100.0)	26 (89.7)	34 (89.5)
Cyclophosphamide	3 (100.0)	3 (100.0)	2 (66.7)	24 (82.8)	32 (84.2)
Purine analog	3 (100.0)	3 (100.0)	3 (100.0)	14 (48.3)	23 (60.5)
Anthracycline	0	2 (66.7)	0	15 (51.7)	17 (44.7)
Bendamustine	0	0	0	5 (17.2)	5 (13.2)

[a]Refractory is defined as not responding to a standard regimen or progressing within 6 months of the last course of a standard regimen.

[b]Four investigators selected "unknown" as the answer to the question "Is patient relapsed or refractory to prior treatment?" If "Unknown" was selected, the site had to confirm patient's eligibility for inclusion in the study.

BID, twice daily; CLL, chronic lymphocytic leukemia; DLBCL, diffuse large B-cell lymphoma; FL, follicular lymphoma; HL, Hodgkin's lymphoma; LPL/WM, lymphoplasmacytoid lymphoma/Waldenström macroglobulinemia; MCL, mantle cell lymphoma; MZL, marginal zone lymphoma; NHL, non-Hodgkin's lymphoma; Q1, first quartile; Q3, third quartile.

Supplemental Table 3. ORR per IRC assessment[a]

IRC Assessments	50 mg BID (N=3)	100 mg BID (N=3)	200 mg BID (N=3)	400 mg BID (N=29)	Total (N=38)
ORR[b], n (%)	1 (33.3)	1 (33.3)	2 (66.7)	12 (41.4)	16 (42.1)
95% CI[c]	0.8, 90.6	0.8, 90.6	9.4, 99.2	23.5, 61.1	26.3, 59.2
CR	0	0	0	0	0
PR	1 (33.3)	1 (33.3)	2 (66.7)	12 (41.4)	16 (42.1)
SD	2 (66.7)	1 (33.3)	0	9 (31.0)	12 (31.6)
PD	0	1 (33.3)	1 (33.3)	4 (13.8)	6 (15.8)
NE	0	0	0	4 (13.8)	4 (10.5)

IRC Assessments	400 mg BID cohort only (N = 29)		
	CLL (N=15)	NHL/HL (N=14)	Total (N=29)
ORR[b], n (%)	8 (53.3)	4 (28.6)	12 (41.4)
95% CI[c]	26.6, 78.7	8.4%, 58.1	23.5, 61.1
CR	0	0	0
PR	8 (53.3)	4 (28.6)	12 (41.4)
SD	6 (40.0)	3 (21.4)	9 (31.0)
PD	0	4 (28.6)	4 (13.8)0
NE	1 (6.7)	3 (21.4)	4 (13.8)

[a]The full analysis set includes patients who received ≥1 dose of acalisib.

[b]ORR is the percentage of patients who had best overall response of complete response or partial response. Patients who do not have sufficient baseline or on-study tumor assessment to characterize response are included in the denominator.

[c]95% exact binomial confidence interval of overall response rate.

BID, twice daily; CI, confidence interval; CLL, chronic lymphocytic leukemia; CR, complete response; HL, Hodgkin's lymphoma; IRC, independent review committee; NE, nonevaluable; NHL, non-Hodgkin's lymphoma; ORR, overall response rate; PD, progressive disease; PR, partial response, SD, stable disease.

Supplemental Table 4. All grade and grade ≥3 adverse events in ≥10% of patients

Adverse events, n (%)	50 mg BID (N=3)		100 mg BID (N=3)		200 mg BID (N=3)		400 mg BID (N=29)		Total (N=38)	
	All	Grade ≥3	All	Grade ≥3	All	Grade ≥3	All	Grade ≥3	All	Grade ≥3
**All AEs**	3 (100)	3 (100)	3 (100)	2 (66.7)	3 (100)	3 (100)	29 (100)	24 (82.2)	38 (100)	32 (84.2)
**Fatigue**	2 (66.7)	1 (33.3)	2 (66.7)	1 (33.3)	3 (100.0)	1 (33.3)	14 (48.3)	1 (3.4)	21 (55.3)	4 (10.5)
**Pyrexia**	0	0	1 (33.3)	0	1 (33.3)	0	12 (41.4)	0	14 (36.8)	0
**Cough**	1 (33.3)	0	0	0	1 (33.3)	0	12 (41.4)	0	14 (36.8)	0
**Diarrhea**	2 (66.7)	1 (33.3)	0	0	2 (66.7)	1 (33.3)	9 (31.0)	3 (10.3)	13 (34.2)	5 (13.2)
**Edema peripheral**	1 (33.3)	0	0	0	3 (100.0)	0	8 (27.6)	0	12 (31.6)	0
**Dyspnea**	1 (33.3)	0	1 (33.3)	0	2 (66.7)	0	7 (24.1)	2 (6.9)	11 (28.9)	2 (5.3)
**Weight decreased**	1 (33.3)	0	0	0	1 (33.3)	0	9 (31.0)	1 (3.4)	11 (28.9)	1 (2.6)
**Rash**	1 (33.3)	0	0	0	0	0	9 (31.0)	4 (13.8)	10 (26.3)	4 (10.5)
**Anemia**	0	0	1 (33.3)	1 (33.3)	0	0	9 (31.0)	4 (13.8)	10 (26.3)	5 (13.2)
**Pneumonia**	2 (66.7)	1 (33.3)	0	0	1 (33.3)	1 (33.3)	5 (17.2)	4 (13.8)	8 (21.1)	6 (15.8)
**Influenza-like illness**	0	0	1 (33.3)	0	2 (66.7)	0	4 (13.8)	0	7 (18.4)	0
**Nausea**	0	0	0	0	0	0	7 (24.1)	0	7 (18.4)	0

**Vomiting**	1 (33.3)	0	1 (33.3)	0	0	0	4 (13.8)	0	6 (15.8)	0
**Abdominal pain**	0	0	0	0	1 (33.3)	0	5 (17.2)	1 (3.4)	6 (15.8)	1 (2.6)
**Urinary tract infection**	0	0	0	0	2 (66.7)	1 (33.3)	4 (13.8)	2 (6.9)	6 (15.8)	3 (7.9)
**Alanine aminotransferase increased**	0	0	1 (33.3)	0	0	0	5 (17.2)	1 (3.4)	6 (15.8)	1 (2.6)
**Aspartate aminotransferase increased**	0	0	1 (33.3)	0	0	0	5 (17.2)	1 (3.4)	6 (15.8)	1 (2.6)
**Dysgeusia**	1 (33.3)	0	0	0	0	0	5 (17.2)	0	6 (15.8)	0
**Insomnia**	0	0	0	0	0	0	6 (20.7)	2 (6.9)	6 (15.8)	2 (5.3)
**Decreased appetite**	1 (33.3)	0	0	0	0	0	5 (17.2)	0	6 (15.8)	0
**Dyspepsia**	0	0	1 (33.3)	0	1 (33.3)	0	3 (10.3)	0	5 (13.2)	0
**Skin infection**	0	0	0	0	1 (33.3)	0	4 (13.8)	0	5 (13.2)	0
**Lung infection**	0	0	0	0	0	0	4 (13.8)	2 (6.9)	4 (10.5)	2 (5.3)
**Upper respiratory tract infection**	1 (33.3)	0	1 (33.3)	0	0	0	2 (6.9)	0	4 (10.5)	0
**Constipation**	0	0	0	0	1 (33.3)	0	3 (10.3)	0	4 (10.5)	0
**Neutrophil count decreased**	0	0	0	0	0	0	4 (13.8)	3 (10.3)	4 (10.5)	3 (7.9)
**Back pain**	2 (66.7)	0	1 (33.3)	0	0	0	1 (3.4)	0	4 (10.5)	0

AEs were classified using MedDRA version 19.0. Patients who experienced multiple events within the same preferred term were counted once. The severity of AEs was graded per the CTCAE version 4.03. Severity grade 1=mild, 2=moderate, 3=severe, 4=life threatening, 5=fatal. AE, adverse event; BID, twice daily

Supplemental Table 5. Laboratory abnormalities reported in ≥15% of patients

Laboratory abnormalities, n (%)	50 mg BID (N=3)		100 mg BID (N=3)		200 mg BID (N=3)		400 mg BID (N=29)		Total (N=38)	
	All	Grade ≥3	All	Grade ≥3	All	Grade ≥3	All	Grade ≥3	All	Grade ≥3
**Increased creatinine**	3 (100.0)	0	1 (33.3)	0	2 (66.7)	0	23 (79.3)	0	29 (76.3)	0
**Decreased neutrophil count**	1 (33.3)	1 (33.3)	3 (100.0)	3 (100.0)	3 (100.0)	2 (66.7)	17 (58.6)	10 (34.5)	24 (63.2)	15 (42.1)
**Hypertriglyceridemia**	2 (66.7)	1 (33.3)	0	0	3 (100.0)	0	15 (51.7)	2 (6.9)	20 (52.6)	3 (7.9)
**Alanine aminotransferase increased**	1 (33.3)	0	0	0	0	0	14 (48.3)	4 (13.8)	15 (39.5)	4 (10.5)
**Increased aspartate aminotransferase**	1 (33.3)	0	1 (33.3)	0	0	0	13 (44.8)	3 (10.3)	15 (39.5)	3 (7.9)
**Increased leukocyte count**	2 (66.7)	2 (66.7)	2 (66.7)	1 (33.3)	0	0	10 (34.5)	6 (20.7)	14 (36.8)	9 (23.7)
**Decreased leukocyte count**	2 (66.7)	2 (66.7)	2 (66.7)	1 (33.3)	0	0	10 (34.5)	6 (20.7)	14 (36.8)	9 (23.7)
**Increased lymphocyte count**	1 (33.3)	1 (33.3)	1 (33.3)	1 (33.3)	2 (66.7)	1 (33.3)	9 (31.0)	8 (27.6)	13 (34.2)	11 (28.9)
**Decreased lymphocyte count**	1 (33.3)	1 (33.3)	1 (33.3)	1 (33.3)	2 (66.7)	1 (33.3)	9 (31.0)	8 (27.6)	13 (34.2)	11 (28.9)

**Decreased hemoglobin**	1 (33.3)	0	0	0	1 (33.3)	0	11 (37.9)	5 (17.2)	13 (34.2)	5 (13.2)
**Decreased platelet count**	1 (33.3)	0	2 (66.7)	1 (33.3)	2 (66.7)	1 (33.3)	8 (27.6)	3 (10.3)	13 (34.2)	5 (13.2)
**Hypernatremia**	1 (33.3)	0	0	0	1 (33.3)	0	8 (27.6)	1 (3.4)	10 (26.3)	1 (2.6)
**Hyponatremia**	1 (33.3)	0	0	0	1 (33.3)	0	8 (27.6)	1 (3.4)	10 (26.3)	1 (2.6)
**Increased alkaline phosphatase**	1 (33.3)	0	2 (66.7)	0	1 (33.3)	0	7 (24.1)	0	11 (28.9)	0
**Hypophosphatemia**	1 (33.3)	0	1 (33.3)	0	0	0	7 (24.1)	4 (13.8)	9 (23.7)	4 (10.5)
**Hypoalbuminemia**	3 (100.0)	0	0	0	0	0	5 (17.2)	0	8 (21.1)	0
**Hyperkalemia**	0	0	0	0	0	0	7 (24.1)	0	7 (18.4)	0
**Hypokalemia**	0	0	0	0	0	0	7 (24.1)	0	7 (18.4)	0

Presented are laboratory abnormalities at worst grade postbaseline.
Grade 0=none, 1=mild, 2=moderate, 3=severe, 4=potentially life-threatening. BID, twice daily.

Supplemental Table 6. Serious adverse events in ≥5% of all patients and all serious infections

SAEs, n (%)	50 mg BID (N=3)	100 mg BID (N=3)	200 mg BID (N=3)	400 mg BID (N=29)	Total (N=38)
Any SAEs	2 (66.7)	1 (33.3)	2 (66.7)	22 (75.9)	27 (71.1)
Dyspnea	0	0	0	2 (6.9)	2 (5.3)
Respiratory failure	1 (33.3)	0	0	1 (3.4)	1 (2.6)
Pyrexia	0	0	0	4 (13.8)	4 (10.5)
Diarrhea	1 (33.3)	0	0	1 (3.4)	2 (5.3)
**All serious infections and infestations by preferred term**					
Pneumonia	2 (66.7)	0	1 (33.3)	4 (13.8)	7 (18.4)
Urinary tract infection	0	0	1 (33.3)	2 (6.9)	3 (7.9)
Lung infection	0	0	0	1 (3.4)	1 (2.6)
Pulmonary sepsis	0	0	0	1 (3.4)	1 (2.6)
Sepsis	0	1 (33.3)	0	0	1 (2.6)
Urosepsis	0	1 (33.3)	0	0	1 (2.6)
Bronchiolitis	0	0	0	1 (3.4)	1 (2.6)
Pneumonia viral	0	0	0	1 (3.4)	1 (2.6)
Appendicitis	0	0	0	1 (3.4)	1 (2.6)
Bronchopulmonary aspergillosis	0	0	0	1 (3.4)	1 (2.6)
Mycobacterial infection	0	0	1 (33.3)	0	1 (2.6)
Arthritis bacterial	0	0	1 (33.3)	0	1 (2.6)
Genital herpes	1 (33.3)	0	0	0	1 (2.6)
Respiratory tract infection	0	1 (33.3)	0	0	1 (2.6)
Erysipelas	0	0	0	1 (3.4)	1 (2.6)

Patients who experienced multiple events within the same preferred term are counted once.
BID, twice daily; SAE, serious adverse event.

Supplemental Figure 1. Forest plot of overall response rate per dose cohort and IRC assessment. BID, twice daily; IRC, independent review committee; LCL, lower count limit; ORR, overall response rate; UCL, upper count limit

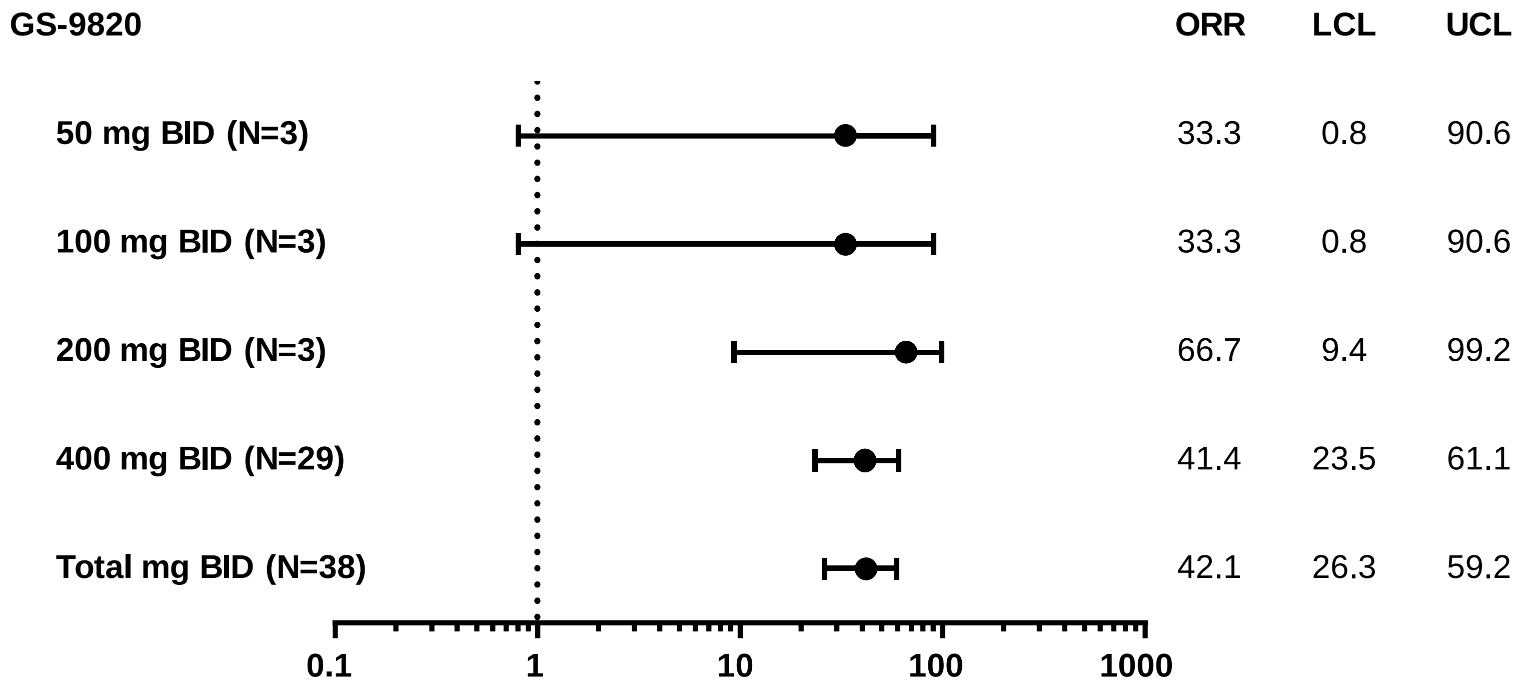

Supplemental Figure 2. Kaplan-Meier curve of progression-free survival per disease type and IRC assessment in 29 patients treated with acalisib 400 mg twice daily. CLL, chronic lymphocytic leukemia; IRC, independent review committee NHL/HL Non-Hodgkin's and Hodgkin's lymphoma, PFS, progression-free survival.

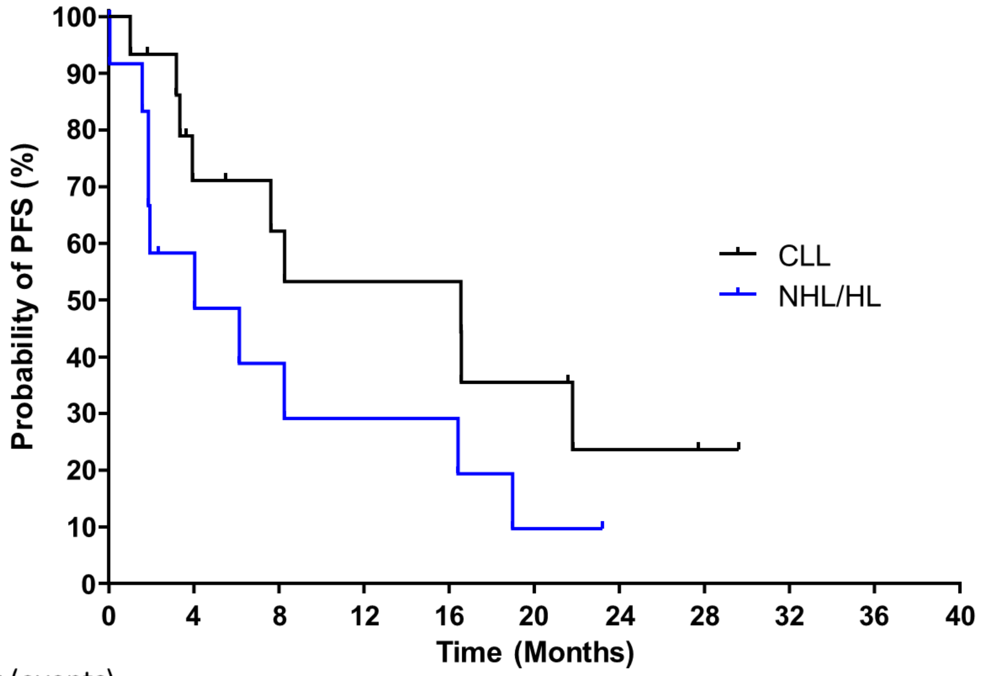

Number at risk (events)

**CLL**	**15 (0)**	**9 (4)**	**7 (5)**	**6 (6)**	**6 (6)**	**4 (8)**	**2 (9)**	**1 (9)**
**NHL/HL**	14 (0)	6 (5)	4 (7)	3 (8)	3 (8)	1 (10)	0 (10)	0 (10)
